# Distributed Fast Self-Organized Maps for Massive Spectrophotometric Data Analysis †

**DOI:** 10.3390/s18051419

**Published:** 2018-05-03

**Authors:** Carlos Dafonte, Daniel Garabato, Marco A. Álvarez, Minia Manteiga

**Affiliations:** 1 CITIC—Department of Computer Science, University of A Coruña, Campus de Elviña s/n, 15071 A Coruña, Spain; marco.antonio.agonzalez@udc.es; 2 CITIC—Astronomy and Astrophysics, University of A Coruña, Campus de Elviña s/n, 15071 A Coruña, Spain; manteiga@udc.es

**Keywords:** remote sensing, computational astrophysics, distributed computing, fast self-organized maps, Apache Hadoop, Apache Spark

## Abstract

Analyzing huge amounts of data becomes essential in the era of Big Data, where databases are populated with hundreds of Gigabytes that must be processed to extract knowledge. Hence, classical algorithms must be adapted towards distributed computing methodologies that leverage the underlying computational power of these platforms. Here, a parallel, scalable, and optimized design for self-organized maps (SOM) is proposed in order to analyze massive data gathered by the spectrophotometric sensor of the European Space Agency (ESA) Gaia spacecraft, although it could be extrapolated to other domains. The performance comparison between the sequential implementation and the distributed ones based on Apache Hadoop and Apache Spark is an important part of the work, as well as the detailed analysis of the proposed optimizations. Finally, a domain-specific visualization tool to explore astronomical SOMs is presented.

## 1. Introduction

Nowadays, information management systems have to be capable of handling enormous volumes of data, so that they can be appropriately captured, stored, managed, and analyzed. This is usually called the era of Big Data, where data scientists need to operate with very large amounts of information by means of Data Mining techniques that allow for running software applications in parallel environments, rather than using standalone applications run on personal computers. This means that typical software packages and algorithms must be adapted towards distributed computing platforms [1,2].

Examples of Big Data environments are the comprehensive all-sky astronomical surveys that capture hundreds of Gigabytes through the on-board sensors, such as the on-going Earth-based Sloan Digital Sky Survey (SDSS) [3], the ESA Gaia space mission [4], and the future surveys planned with the Large Synoptic Survey Telescope (LSST) [5] currently under construction. Gaia is one of the most promising missions of the ESA, and it is expected to become a milestone in Astronomy. Its main objective is to provide accurate positional and kinematical information (position, parallax, proper motion, and radial velocity) of all stars in the Milky Way up to a limiting apparent visible magnitude close to G = 21, with G being the light passband of Gaia photometric instrument [6,7]. Such a complete, non-biased map of the Galaxy will allow researchers to study the properties of the different stellar populations and will provide a reliable picture of the past history of star formation and chemical and dynamic evolution. However, deriving such information from anonymous stars is not very useful, and this is why Gaia is equipped with two low-resolution spectrophotometer sensors: one for the blue wavelengths (BP) and the other for the red wavelengths (RP), which together provide the spectral energy distribution (SED) of all the observed objects from 330 to 1050 nm, sampled with a dispersion varying between 4 to 32 nm pixel${}^{-1}$ [6]. The main physical properties of each individual star observed by Gaia can be derived from their SEDs by the use of different algorithms based, for instance, on statistical or pattern matching approaches.

**Figure 1:**
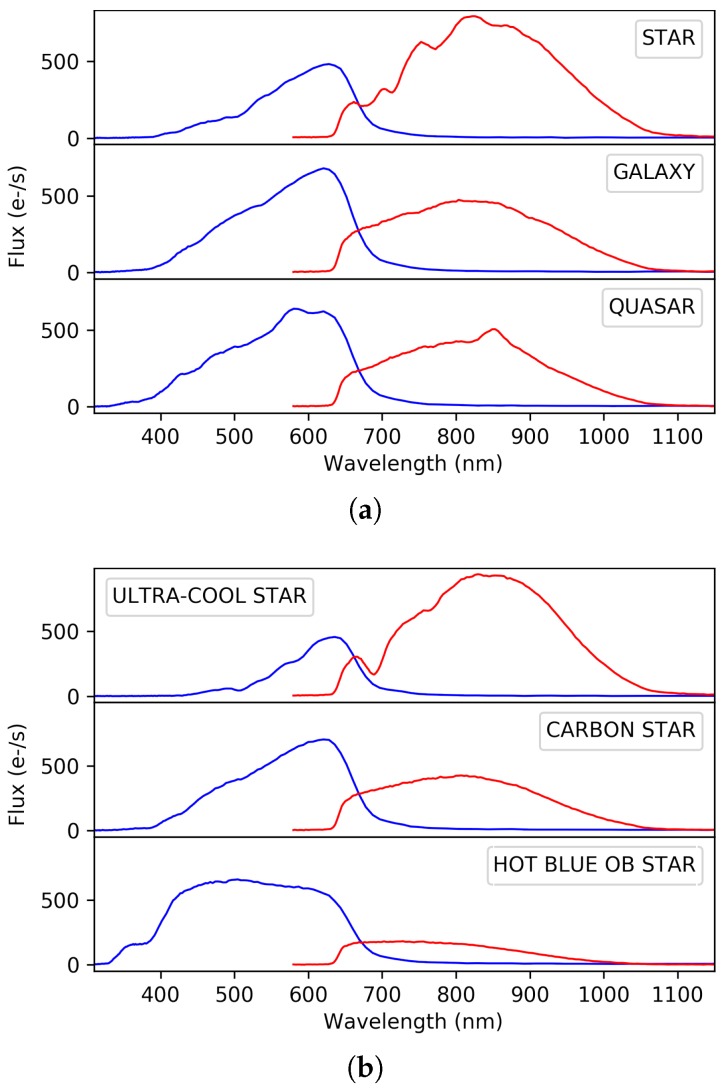
Examples of simulated Gaia spectrophotometric data: Blue Photometer (BP) and Red Photometer (RP). (**a**) simulated Gaia BP/RP data for different object types; (**b**) simulated Gaia BP/RP data for different star types.

Gaia tracks not only stars but also extragalactic objects, such as galaxies and quasars. Because of the distinctive features in their SEDs, the photometric observations obtained with the blue and red sensors will allow for the classification of the sources among the different astronomical object types: single or binary stars, white dwarfs, galaxies, and quasars. Figure 1a shows examples of typical BP/RP spectra for different astronomical objects. Obviously, most of the measurements will correspond to stars, and the combined use of astrometry and photometry from the BP and RP sensors are expected to provide information to accurately derive their astrophysical characteristics, such as effective temperature, surface gravity, and chemical composition. Figure 1b shows BP and RP synthetic photometry for several extreme type stars from very cold and faint ultra-cool stars, chemically anomalous carbon stars, to massive and blue large stars (type O – type B). Once the astrophysical parameters are determined, the use of models will allow us to determine stellar ages and masses and subsequently describe the chemical and dynamical evolution of the Galaxy over a wide range of distances, which is the ultimate objective of the Gaia mission.

The satellite was launched in December 2013, and it started its five years of routine operations in June 2014. Since then, the spacecraft has been gathering approximately 40 Gigabytes every day from the on board instruments, building the widest and most complete census of the Galaxy, sampling astrometric properties and spectral energy distributions of 1.3 billion stars, and turning into the most accurate survey ever made [8]. Taking into account that the spacecraft will observe these objects over 75 epochs on average, the volume of the final Gaia database is expected to be around a Petabyte, becoming a challenge in Computational Astrophysics. Such a catalog will contain calibrated measurements from all Gaia instruments (integrated magnitudes in different filters, spectral energy distributions, parallaxes, proper motions, etc.), as well as estimated astrophysical quantities. This processing and analysis is the main goal of Gaia Data Processing and Analysis Consortium (DPAC), an international scientific network composed of around 450 scientists and engineers, which was promoted in 2005 by the ESA. The consortium is arranged in a hierarchical structure composed of nine coordination units (CU), which are responsible for a part of the whole data processing effort, and six data processing centers (DPC), where the information is actually processed by software algorithms; the latter are designed and implemented by a number of work packages (WP) or modules related to a certain CU according to their purpose. The final Gaia catalog is foreseen for 2023; however, some intermediate releases are published in the meantime: the first one took place in September 2016 [4,9], whereas the second one took place in April 2018.

**Figure 2:**
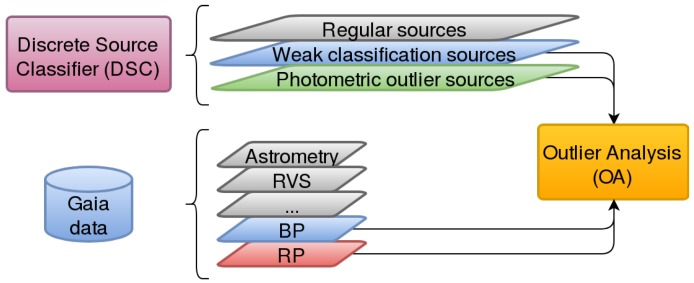
Outlier Analysis (OA) module context: processes Gaia observed data that have been classified as outliers by the Discrete Source Classifier (DSC) package.

This paper is framed in the Astrophysical Parameters Inference System (Apsis, CU8) [10], and the Catalog Access (CU9):

1. Apsis is devoted to the classification of all the observed sources [11,12,13] by means of several work packages enclosed within this CU, the Discrete Source Classifier (DSC) [13] being the main one. It processes the whole Gaia dataset in order to classify the sources into a known astronomical type (star, quasar, galaxy, etc.) by means of Support Vector Machines (SVM) [14], tagging as outliers those sources that do not appropriately fit these models. The rest of the modules are aimed at more specific classification tasks for subgroups of sources that satisfy certain requirements.
  This article is associated with one of these packages, Outlier Analysis (OA, Figure 2), which is devoted to the analysis, using unsupervised Artificial Intelligence methods on spectrophotometric data (BP/RP spectra), of those sources tagged by the DSC module [13] as outlier sources because either they cannot be fitted into any of the models used by DSC so they are considered photometric outliers, or they cannot be classified with enough probability, i.e., weakly classified sources.
  These outlier sources are expected to be of the order of 10% of the whole Gaia dataset, i.e., approximately $10^{8}$ sources (100 Terabytes), which will be processed by the OA module in order to throw some light on their nature, pretending to provide not only plain astronomical object types (stars, ultra-cool dwarfs, white dwarfs, planetary nebulae, quasars, and galaxies), but also sub-types [11].
2. The Catalog Access aims to design and implement the Gaia Archive, providing tools for the astronomical community in order to access the Gaia catalog, visualize data, or even perform data processing tasks. A Data Mining tool based on the OA module will be published, so that scientists can run their own unsupervised analysis on Gaia data. Finally, a visualization tool (Section 4) will be also released to ease the post-analysis stage for OA-based results.

Dealing with outlier sources is a complex task because they may be damaged observations or even objects whose nature is completely unknown, so the information available about them does not have a direct interpretation. In addition, the high dimensionality of the data, which always happens when working with spectrophotometric data, makes the analysis even tougher. According to these issues, two approaches are typically used to analyze outliers:

1. Dimensionality reduction lowers the number of features that is used to explore the data, making the process lighter and therefore faster without losing too much information. Principal Component Analysis (PCA) [15] and Linear Discriminant Analysis (LDA) [16] are the most popular algorithms for dimensionality reduction.
2. Clustering organizes objects into a number of groups with no prior information, just according to the nature of the data. There are many different algorithms available, and they must be carefully selected according to the particular problem under study [17].

Therefore, an algorithm that combines both features would be a great approach to analyze outliers, and this is what we achieved using Self-Organized Maps (SOM) [18,19], and an Artificial Neural Network that projects the input data into a two- or three-dimensional grid, which can be intuitively explored using a dedicated visualization tool. Each processed source belongs to a certain cluster according to its nature, and each cluster has a prototype that represents all the data grouped in such a cluster. The prototypes are adapted over an iterative learning or training procedure that starts from a random distribution of the clusters and finalizes with an ordered representation of the data. There are several variants of the learning algorithm proposed by Kohonen in 1982 [18] that have been empirically found to perform really well in Astronomy [20,21,22,23,24,25,26].

The enormous volumes of information collected in the Gaia mission require the applied algorithms to analyze such data to be adapted in order to process them using a reasonable amount of time and resources. In order to conduct some performance tests a Gaia-based dataset is described in Section 2. Then, we present in Section 3 a custom version of Kohonen’s batch algorithm for optimization, and distributed computing implementations for Apache Hadoop [2] and Apache Spark [1] are proposed in Section 3.1. In addition, it is imposed that all Apsis algorithms are compliant with the System of Accommodation of Gaia Algorithms (SAGA) framework [27,28], an Apache Hadoop abstraction specifically developed for the Gaia software processing pipeline in order to execute the algorithms at the Centre National d’Études Spatiales (CNES) in Toulouse, France. Consequently, the OA module has been also implemented in SAGA, according to its scheme (Section 3.2). Regarding the analysis of the results, a domain-specific visualization tool is introduced in Section 4, and a performance comparison among all the algorithm versions is made in Section 5. Finally, some conclusions and future lines of work are discussed in Section 6 and Section 7, respectively.

## 2. The Gaia Data

All the information gathered by Gaia and its on-board sensors will be published along with the Gaia catalog by means of several incremental Data Releases. By this time, the first Gaia Data Release was delivered in September 2016 and the second one in April 2018, but no Gaia spectrophotometric data is included in these releases. It is expected that such data is added to the catalog during the third Data Release, foreseen for 2020.

Although the algorithm is being internally tested, using real Gaia data for validation purposes, DPAC explicitly prohibits the publication of any real data or results based on them before its official publication in the Data Releases. Therefore, the data used and the results presented here are based on semi-empirical observations from Sloan Digital Sky Survey (SDSS) DR7 [29]; it contains 10,125 sources labeled as “unknown” by the SDSS spectroscopic classification system, which were adapted to the BP and RP photometric format using the Gaia Object Generator (GOG) [30,31]. GOG was designed by DPAC in order to simulate the end-of-mission catalog, including observational errors, with the objective to probe the performance of the analysis algorithms under development. In [31], a full description of GOG is provided, including the models assumed for the performance of Gaia spectrophotometers. In the case of simulated spectrophotometry, GOG transforms “true” object properties, i.e., the SED obtained from a model or from suited spectroscopic observations, into “Gaia observed” fluxes that have an associated error that depends on the object’s properties, Gaia’s BP/RP instrument response, and the type and number of observations made.

The proposed dataset has been studied in detail in [21]: it is a very representative sample of outlying data for the Gaia Outlier Analysis task due to the nature of these sources, which are mainly very faint and damaged observations. However, in order to test the performance and scalability of the OA module on big datasets, such as that of Gaia, it would be necessary to work with different datasets, from a small one (i.e., the one presented above) to huge datasets containing millions of sources. Hence, we decided to take the SDSS semi-empirical dataset as the baseline and repeat it several times so as to build larger datasets whose dimensionality is of the order of those expected for the upcoming Data Releases. This does not really influence the tests conducted, as long as the quality of the clustering is not being assessed in this work. For further details on clustering quality provided by the algorithm, see [21].

### BP/RP Spectra Preprocessing for Outlier Analysis

Before working with the data, they must be treated in order to make them compliant with the specifications of the algorithms. Furthermore, applying an appropriate preprocessing may significantly increase the overall performance in terms of both the quality of the results and the processing time.

**Figure 3:**
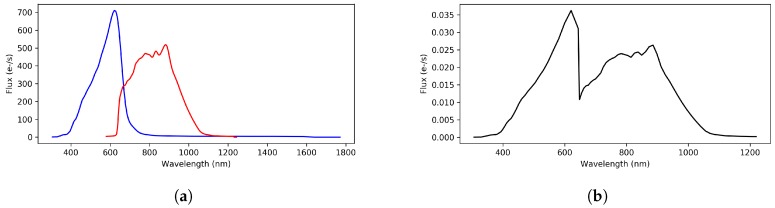
Gaia spectrophotometric data preprocessing for the Outlier Analysis (OA) module: the spectra provided by the Blue and the Red Photometers (BP/RP) are sanitized, downsampled, clipped, and joined into a single spectrum. Finally, the extinction is treated by applying a Cardelli extinction model [32] and the flux is normalized to unit area. (**a**) original Gaia BP/RP spectra; (**b**) preprocessed spectrum for OA.

For the Outlier Analysis module, a preprocessing stage on BP/RP spectra was defined in order to enhance the results provided by the SOM. Among all the configurations explored, the following steps, which are summarized in Figure 3, were empirically found to offer a better performance:

1. The low signal-to-noise ratio (SNR) pixels that lie on the extremes of BP/RP spectra are discarded due to the low efficiency of the passbands (less than 5%). It may be remarked that this is a common step for almost all the CU8 algorithms.
2. The BP/RP spectra are sanitized, interpolating those pixels where the passband efficiency is acceptable and whose flux value is missing or wrong, such as negative values.
3. GOG data is oversampled, and a downsampling is done on the given BP/RP spectra in order to reduce their dimensionality from 180 pixels to 60 pixels. Despite losing some information, it does save up to 60% of processing time and the clustering quality is not significantly affected by this decision (less than 1% effect in the tests conducted). Apart from OA, a number of modules are also taking similar approaches to speed up their execution.
4. Both BP and RP spectra are joined into a single spectrum, removing the overlapping region to avoid redundant wavelengths at a cut point, which is empirically determined by a domain expert according to the response of the satellite’s instrument. Currently, the same cut point is being used for all the objects, although this may vary in the future since the flux and wavelengths calibration for the spectrophotometry is not final yet.
5. The Cardelli extinction model [32] is applied to the joint spectrum in order to minimize the impact of interstellar reddening on the classification.
6. Finally, the treated spectrum is scaled to fix its area to one unit, so that sources with different brightness can be compared using similarity distance functions on their spectra:
  (1) $$F_{i}^{\prime }=\frac{F_{i}}{\sum_{i\in S}\left(F_{i}\right),}$$
  where $F_{i}$ is the flux of a spectrum in band *i*, and *S* represents the bands of the spectrum.

## 3. Analyzing Gaia Outliers by Means of Self-Organized Maps

Self-Organized Maps (SOM) [18,19] were chosen because of their ability to process high-dimensional data, such as the Gaia spectrophotometric data, and because they can be explored by the domain experts by means of a dedicated visualization tool for astronomical SOMs (Section 4). However, despite being a general Machine Learning method, SOM must be configured or adapted according to the domain.

**Figure 4:**
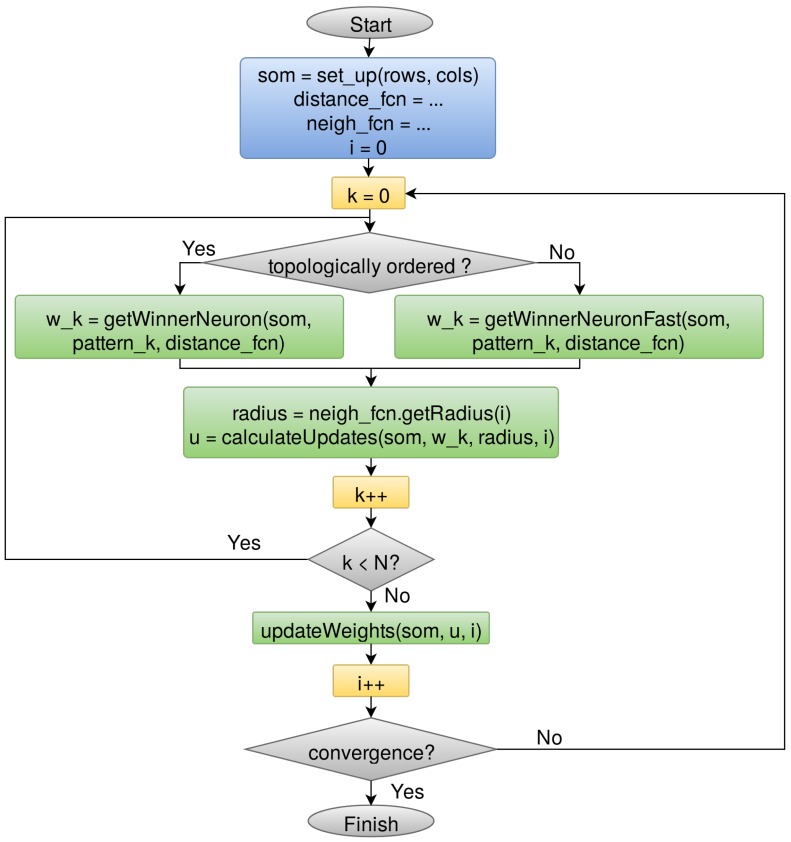
Fast Self-Organized Map (SOM) batch learning algorithm [20]: it starts with a random initialization of the neurons, followed by an adaptation over an iterative process. This includes, for each input source, determining the winner neuron using a distance function and calculating the weights update needed for the winner neuron and the neighbors involved. Once the whole dataset is processed, the weights update is finally committed to the SOM and the convergence criteria are analyzed to check whether it needs to continue iterating or not.

**Figure 5:**
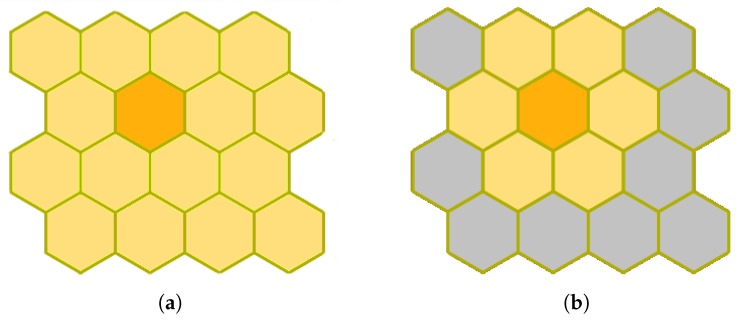
Differences in Self-Organized Map search spaces to determine a winner neuron for the current iteration. The search spaces are highlighted in yellow, whereas the winner neuron in the previous cycle is colored in orange. (**a**) regular SOM: the winner neuron is searched within the whole map; (**b**) fast SOM: The search space is restricted to the immediate neighborhood of the previous winner.

The Outlier Analysis module will handle approximately $10^{8}$ sources, around 100 Terabytes, so it must be parallelizable in order to make its computation feasible. We opt for the batch method (Figure 4) because it allows for parallelization at iteration level by processing the whole dataset at once before updating the SOM prototypes [33]. This makes the algorithm even more stable because it does not take into account the order within the dataset. On the contrary, the online family does an observation-by-observation processing and updating of the map, making its parallelization unfeasible. In addition, the Kohonen’s batch algorithm was sped up by the use of FastSOM [20], that, once the map has become topologically ordered after the first iterations, reduces the search space for determining the neuron that better resembles each input source (Figure 4). These neurons are usually called winner neurons and they are found by means of a similarity distance function, such as the Euclidean or Manhattan distances. In spite of slightly decreasing the quality of the results (less than 5% for reference datasets), FastSOM allows us to speed up the algorithm by reducing the number of distance computations, looking for the winner neuron within the immediate neighborhood of the previous winner (Figure 5b) instead of exploring the whole map (Figure 5a).

The metric has a high impact on both the execution time of the algorithm (since distances are evaluated many times) as the quality of the classification. Therefore, there must be a balance between both items. It was found that the squared Euclidean distance achieves a slightly better performance than others, such as the Chebyshev or Manhattan distances. Another parameter that must be selected is the neighborhood function, which determines how the neighbors of the winner neurons are affected during the weight update calculation. It determines how many neighbors are considered (usually called radius), and it must progress from a large value during the first iterations (denoted by $\sigma_{0}$) to zero (that is the winner neuron itself), progressively shrinking as the iterations succeed (controlled by a parameter denoted by *T*, the neighborhood decay). A better performance was empirically obtained for the Gaussian neighborhood function, and $\sigma_{0}=30$ and T=50.

There are two remaining hyper-parameters of the SOM that must be set up: the dimensions of the map, and the convergence criteria. Since the results of the OA module must be explored by the scientific community, a two-dimensional grid was preferred for visualization purposes, and the number of rows and columns was empirically determined using the Elbow method [34]. It studies how much information is gained by adding more clusters until a significant drop is obtained. In this case, the optimal configuration is found to be around 900 clusters arranged in a 30×30 grid. Finally, it can be considered that the algorithm converges when it is stable and no further mutation is required on the prototypes. However, if such a condition is not reached, a maximum of 500 iterations is allowed to guarantee that it stops at some point.

### 3.1. A Parallel Self-Organized Maps Learning Algorithm

The main objective of distributing the SOM training stage is to implement a version of the algorithm capable of handling vast amounts of high-dimensional data in order to process and analyze them in a reasonable period of time. A wide variety of distributed computing frameworks are available, from low-level solutions, such as Message Passing Interface (MPI) [35], to high-level abstractions such as Apache Hadoop [2] or Apache Spark [1]. In order to process the Gaia data, both Apache frameworks were chosen by DPAC coordination units 8 and 9, respectively.

The actual implementation is mainly based on the Map-Reduce paradigm [36], which is a general-purpose parallel programming model intended for spreading the workload over a number of machines arranged in a cluster. Firstly, during the map stage, the data are distributed across the machines, so that each chunk is processed individually. Once the map tasks are over, the reduce phase takes place, gathering and processing all the outputs produced by the map tasks. This paradigm is highly configurable and flexible and further details can be found in [1,2,36].

**Figure 6:**
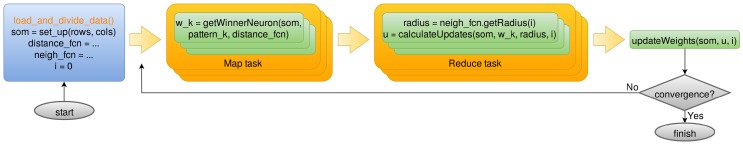
Map-Reduce design for Self-Organized Maps: the map task determines the winner neuron for each input source, whereas the reduce task calculates partial weight updates for each winner neuron; the driver commits the weight updates and takes care of the learning process.

The behavior of such tasks can be controlled by user-defined functions and driver programs that take care of the overall algorithm. For the Map-Reduce implementation of the SOM, the map task computes the winner neuron for each input preprocessed source, while the reduce task calculates partial prototype updates for each winner neuron according to the sources that belong to it, as is illustrated in Figure 6. The driver program takes care of the iterative process itself, as well as it merges and commits the weight updates to the SOM. In the case of Apache Hadoop [2] the implementation is straightforward, defining the map and reduce functions as stated above. On the contrary, Apache Spark [1] is not really based on the Map-Reduce paradigm, but on Resilient Distributed Datasets (RDDs). An RDD is an in-memory distributed collection of observations, which can be transformed or operated in order to compute a result. Therefore, although the map task can be implemented directly using Spark RDD’s equivalent function, the reduce task needs to be translated into a Spark RDD’s combine procedure. In addition, the driver is also optimized by using the built-in Spark RDD’s reduce procedure.

### 3.2. Integration into SAGA Software Pipeline

**Figure 7:**
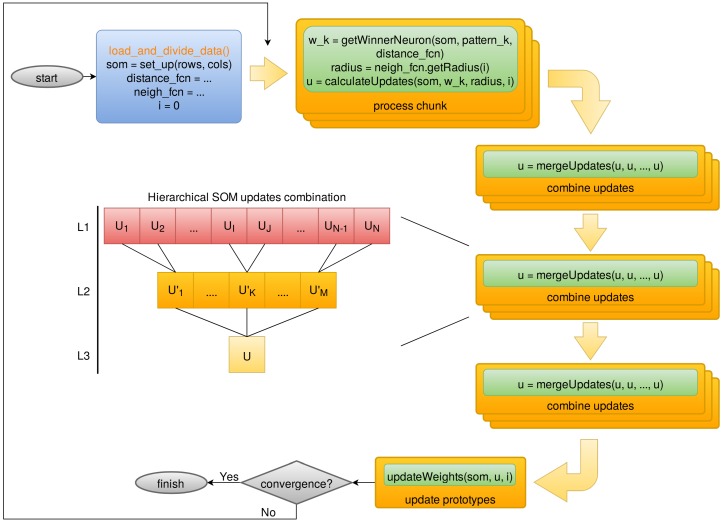
System of Accommodation of Gaia Algorithms (SAGA) facades design for Self-Organized Maps (SOM) that will run at the Centre National d’Études Spatiales (CNES).

The Outlier Analysis algorithm will be ultimately executed under the System of Accommodation of Gaia Algorithms (SAGA) platform [27,28], which is actually a framework on top of Apache Hadoop to handle all Gaia software executed at the Centre National d’Études Spatiales (CNES). Hence, the OA algorithm has to be compliant with SAGA requirements and constraints, and consequently the parallel design discussed in Section 3.1 was adapted towards SAGA’s facade-based paradigm (Figure 7), rather than the previous Map-Reduce one. These facades are directly handled by SAGA and translated into several Apache Hadoop jobs, with little control from the developer side.

The approach used for SAGA is slightly different from the pure Map-Reduce one, as is shown in Figure 7. Firstly, the observations are divided into chunks, which are processed individually in the “process chunk” step, computing partial prototype updates for each chunk. Then, these updates are merged into a single one using a hierarchical procedure implemented through “combine updates” steps. Finally, the merged update is committed to the SOM in the “update prototypes” step.

It must be noticed that such a design is intended for SAGA and, despite the fact that it is possible to translate it into a pure Apache Hadoop or Apache Spark solution, it is certainly an arduous task as it requires to implement some high level operations to handle data that are provided by the SAGA framework. In any case, SAGA design has been found to consume many more resources than the others due to the overhead introduced by SAGA itself, as will be discussed in Section 5.

## 4. A Visualization Tool to Explore Astronomical Self-Organized Maps

**Figure 8:**
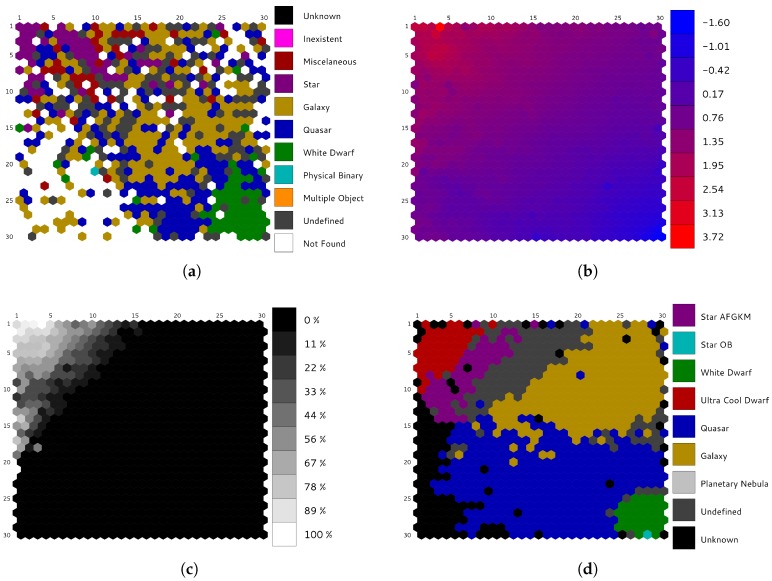
Specialized views for astronomical Self-Organized Maps supported by the visualization tool. (**a**) catalog labels: representative object type for each cluster using a Simbad external catalog; (**b**) color distribution: magnitude differences on blue and red wavelengths for Gaia BP/RP spectrophotometry; (**c**) category distribution: percentage of ultra-cool dwarfs populating the neurons; (**d**) template labels: labels determined using a template matching method based on Gaia simulations.

**Figure 9:**
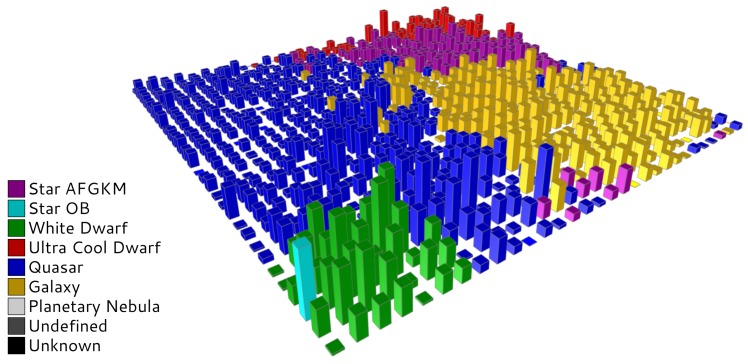
Combined view: the color related to each bar indicates the representative label determined by a template matching procedure on Gaia simulated data, whereas the height of the bar represents the number of elements populating such a cluster.

Self-Organized Maps are meant to be analyzed by experts in the field, i.e., astrophysicists, and this procedure can be really arduous without any help, since the SOM itself is just a large amount of numbers that must be somehow interpreted. One of the typical ways to explore SOMs is to visualize them using partitioning schemes, which, in this case, are mainly created using pre-built templates based on external catalogs and synthetic data. In order to display this information, a dedicated visualization tool for exploring SOMs built on astronomical data has been proposed [37]. Such a tool provides several SOM representations that display the inner structure of the data and the relationships they contain, so that any user can visualize not only the classical hits or u-matrix views, but also more sophisticated ones:

- Catalog labels (Figure 8a) shows the most probable astronomical class associated with each cluster, according to an offline cross-match performed on external astronomical databases, such as Simbad [38], using the web service provided by the database to perform a radial search on the sources’ celestial positions (right ascension and declination), and keeping just the closest one.
  The application can handle different cross-matches simultaneously, so that the user can visualize and compare them.
- Color distribution (Figure 8b) displays how the sources are distributed among the neurons according to their color. Magnitude differences at blue and red wavelengths are depicted in the map using a color gradient.
- Category distribution (Figure 8c) represents a particular type of astronomical object (i.e., stars, white dwarfs, quasars, etc.), displaying how such sources are distributed among the neurons.
- Combined visualizations (Figure 9) allow the user to explore different views by means of three-dimensional graphs, where a qualitative property (labels, category, etc.) is shown as the baseline, and a quantitative property (hits, distance, etc.) is represented as height bars. Information displayed from different perspectives allows the user to discover new relationships within the data.
- Template labels (Figure 8d) allow the user to observe the representative label associated with each neuron. These classes are determined by a template matching procedure on different pre-built model sets, and the user can select which one to display. In Figure 8d, a set on reference models based on Gaia simulations are displayed on top of the original dataset described in Section 2.

This tool offers many other detailed views to analyze SOMs. For example, it is possible to explore the content of a certain neuron, studying the spectra of the sources that belong to it, and some statistics about their astrophysical parameters. Additionally, it is possible to perform a cross-match with external catalogs, to work with different labels based on template matching or external catalogs, and to communicate with other astronomical tools through the Simple Application Messaging Protocol (SAMP) [39].

## 5. Performance Evaluation

Self-Organized Maps have been proved to be a powerful tool for clustering analysis on astronomical datasets [21,25,26]. Although it is not the main goal of the present work, a brief summary about the scientific results obtained for such a dataset is presented here: the SOM succeeded in ordering the sources and grouping them by similarity of their SED. Different object types are placed in clearly different regions in the map (Figure 8d and Figure 9). Despite working with outlier observations, only 15% were misclassified (“undefined”) or not classified at all (“unknown”) by our SOM. Due to the constraints imposed by DPAC, it is not possible to publish results based on any real data; even so, during the first tests conducted for validation purposes the scientific results produced by the algorithm were meaningful and scientifically consistent.

Now, setting aside the scientific results produced by the SOM, the performance obtained for the different implementations proposed in Section 3 will be assessed, running them over exactly two hundred iterations. Consequently, a common environment is defined in Table 1 in order to conduct all those tests under similar conditions. It must be noticed that the SAGA framework is hosted by CNES in Toulouse (France) and it is exclusively intended for official Gaia data processing, so we have limited access to it and only the SAGA compliant algorithm can be tested during the validation and operations runs scheduled by DPAC. The Apache Hadoop and Apache Spark implementations are tested using a much more modest local cluster located at our research laboratory at the University of A Coruna.

**Figure 10:**
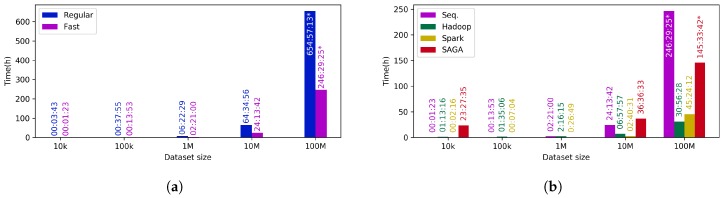
Performance comparison among the different Self-Organized Maps’ implementations described in Section 3. (**a**) speed up obtained by the FastSOM compared to the regular one using a sequential approach; (**b**) scalability of the FastSOM algorithm for the proposed implementations. * These values were estimated.

Firstly, the impact of the use of the FastSOM approach instead of the regular one is assessed. It can be clearly observed in Table 2 and Figure 10a that the FastSOM approach provides a noticeable speed up, reducing execution times up to 60%. It must be also noticed that the larger dataset is processed or the larger number of iterations are completed, the more speed up is obtained with respect to the regular algorithm. This behavior is caused by the reduction of the winner neuron search space (Figure 5), and its impact becomes much greater as the number of winner neuron searches is increased (i.e., more data, or more iterations).

Taking into account the considerable speed up achieved by the FastSOM method, and the insignificant loss of precision, which was found to be less than 5% using a reference dataset (see [20] for further details), we come to the conclusion that it is worth using such an optimization to analyze outliers in the Gaia mission. Therefore, from now on, the performance and scalability results are just shown for the FastSOM algorithm (Table 3 and Figure 10b).

It can certainly be observed that the application of distributed algorithms for processing small datasets provides lower execution times than using a sequential approach. Such a behavior is due to the frameworks internal operations (i.e., initialization, communication among the nodes, finalization, etc.), which consume a certain amount of resources apart from the actual algorithm computation. A threshold can be identified for each of the parallel implementations so that it is worth its execution rather than the sequential one. For the studied dataset, the Apache Hadoop threshold can be set up at around a million sources, whereas Apache Spark can use a lower value, close to fifty thousand sources. In the case of SAGA, the overhead is considerably higher, so that it requires much larger datasets at around a hundred million objects to benefit from such platform.

In general terms, the Apache Spark based algorithm tends to be significantly faster than the Apache Hadoop one for small and medium size datasets, but, for much larger datasets, it becomes less beneficial. The underlying cause of this behavior is the way these frameworks handle data internally: Apache Hadoop makes an extensive use of disk, whereas Apache Spark runs mainly in memory. Thus, for small datasets, the usage of memory rather than disk makes Apache Spark much faster, similar to the sequential implementations, but when the dataset becomes huge, it turns unstable and quite dependent on the underlying platform resources, requiring a fine-tuning of the tasks. In return, Apache Hadoop is more stable when it has to deal with enormous volumes of data, as it uses disk storage whose capacity is usually larger and cheaper than memory, but of course it penalizes the overall performance.

Regarding SAGA-CNES runs, it is necessary to mention and to make clear that it is not possible to run the wide benchmark used in the local cluster, since it is a shared platform for the entire Gaia software pipeline across several coordination units and, therefore, it can only be used to analyze real Gaia data for operations and validation purposes, as scheduled by DPAC. The performance observed using SAGA was not as good as expected, as it was found to add a considerable overhead due to the initialization of the processes and input/output internal operations. However, it must be noticed that the runs under consideration were the first ones for the OA module since its integration, and it is expected to evolve and improve the execution times once the platform configuration is appropriately tuned. Additionally, CNES is continuously working to improve SAGA implementation and new patches are expected to enhance its overall performance.

In any case, the enormous computational power provided by the SAGA-CNES cluster is expected to allow the algorithm to scale well on huge datasets. This scalability is achieved by using chunks, so that the chunk size must be appropriately tuned in order to make use of all the CPUs of the cluster, taking advantage of the underlying computational power. According to the estimations made in the present work, it would be possible to process a hundred million sources in approximately six days (considering the worst case scenario based on a real 28 million objects execution, which took two days), accomplishing the outlier analysis task in very reasonable terms.

## 6. Conclusions

The extensive and complex datasets that are being gathered these days in Astronomy as well as in other fields of research led to a change in the perspective of algorithm and software development towards distributed computing methodologies that can appropriately handle such volumes of data. This is the case of the Gaia mission that is collecting approximately 40 Gigabytes per day and, therefore, it requires a parallel implementation for all the related software developments.

This article addresses the design and implementation of an unsupervised clustering algorithm based on Self-Organized Maps (SOM) in order to conduct an analysis of outlier sources in the Gaia mission through the Outlier Analysis (OA) module. To this purpose, the algorithm was implemented using the frameworks chosen by DPAC to implement their algorithms: Apache Spark and an ad hoc framework based on Apache Hadoop.

Regarding the proposed design (Section 3) and the performance obtained by the algorithm (Section 5), the following conclusions can be highlighted:

- The use of the FastSOM algorithm can considerably speed up the execution, saving up to 60% of the run time compared to the regular implementation, without losing precision (less than 5% for reference datasets).
- A scalable and distributed design was achieved for the SOM learning algorithm, allowing for the analysis of very large data volumes in a reasonable term (Section 5).
- The Apache Spark implementation was found to be really beneficial for small and medium size datasets. However, for enormous volumes of data the intensive memory usage causes the algorithm to become unstable and eventually resource dependent, needing a fine-tuning to avoid excessive memory consumption that could make the algorithm crash at some point.
- The Apache Hadoop version is capable of handling and processing huge datasets in reasonable times, providing a scalable and stable solution for processing vast volumes of information.
- The OA module has been successfully integrated into the CU8 software pipeline (SAGA), and it is planned to produce its first scientific results for the third Gaia Data Release around 2020. According to the tests conducted during the validation stage, OA is expected to take approximately six days to be executed over a hundred million sources (a 10% of the whole Gaia dataset).
- The Apache Spark implementation will be available to the astronomical community as a DPAC CU9 Data Mining tool in order to conduct their own analysis on Gaia data.
- Finally, a visualization tool to explore astronomical SOMs will be published along with the Gaia Data Releases, so that the OA module results, as well as other samples of Gaia data processed using the CU9 Data Mining tool, can be further analyzed by the community.

## 7. Future Work

Although the proposed algorithm is already integrated into the Gaia software pipeline in order to produce scientific results in the upcoming third Gaia Data Release, some key points that could improve this work have been identified:

- Regarding the quality of the scientific results, alternative preprocessing stages are being studied, as well as different postprocessing methods oriented to the visualization tool (Section 4).
- The overhead caused by SAGA internal operations is expected to be significantly reduced in the upcoming SAGA implementations, so that it will speed up the execution of the modules, including the OA module.
- In order to improve the performance of the algorithm, some very promising CPU/GPU mixed computing tests have been conducted for an implementation based on Nvidia Compute Unified Device Architecture (CUDA) (Santa Clara, CA, USA) [40]. Although SAGA does not support GPU computing, this paradigm may be suitable for both Apache Hadoop and Apache Spark and it is currently being studied.
- Our clustering analysis tool based on Self-Organized Maps is being applied to study outlier sources in the Gaia mission, but it could be used to analyze other complex databases, even from other domains. Very promising results have been found for intrusion detection over communication networks, as well as for user profile identification in online marketing environments.

## Figures and Tables

**Table 1:** Execution environment used for the tests. All these machines are under Oracle Java 8.

	# of Cores	Memory (GB)
Local single machine	32	128
Local cluster	104	392
SAGA-CNES cluster	∼1100	6050

**Table 2:** Performance comparison between regular and fast SOM algorithms using a sequential approach executed in a single local machine (hh:mm:ss).

	10 k	100 k	1 M	10 M	100 M
Regular	00:03:43	00:37:55	06:22:29	64:34:56	654:57:13 ${}^{*}$
Fast	00:01:23	00:13:53	02:21:00	24:13:42	246:29:25 ${}^{*}$

* These values were estimated.

**Table 3:** Time measurements to study the scalability of the FastSOM algorithm using the proposed implementations (hh:mm:ss).

	10 k	100 k	1 M	10 M	100 M
Local Sequential	00:01:23	00:13:53	02:21:00	24:13:42	246:29:25 ${}^{*}$
Local Apache Hadoop	01:13:16	01:35:06	02:16:15	06:57:57	30:56:28
Local Apache Spark	00:02:16	00:07:04	00:26:49	02:40:31	45:24:12
SAGA-CNES	23:27:35	-	-	36:36:33	145:33:42 ${}^{*}$

* These values were estimated.
